# CRALBP is a Highly Prevalent Autoantigen for Human Autoimmune Uveitis

**DOI:** 10.1155/2007/39245

**Published:** 2007-11-05

**Authors:** Cornelia A. Deeg, Albert J. Raith, Barbara Amann, John W. Crabb, Stephan R. Thurau, Stefanie M. Hauck, Marius Ueffing, Gerhild Wildner, Manfred Stangassinger

**Affiliations:** ^1^Institute for Animal Physiology, Ludwig-Maximilians University, Veterinärstraße 13, 80539 Munich, Germany; ^2^Cole Eye Institute and Lerner Research Institute, Cleveland Clinic, 9500 Euclid Avenue, Cleveland, OH 44195, USA; ^3^Section of Immunobiology, Department of Ophthalmology, Ludwig-Maximilians University, Mathildenstraße 8, 80336 Munich, Germany; ^4^Institute of Human Genetics, GSF National-Research Center for Environment and Health, Ingolstaedter Landstraße 1, 85764 Neuherberg, Germany; ^5^Institute of Human Genetics, Technical University of Munich, Trogerstraße 32, 81675 Munich, Germany

## Abstract

Cellular retinaldehyde binding protein (CRALBP) is an autoantigen in spontaneous equine recurrent uveitis. In order to test whether CRALBP contributes to human autoimmune uveitis, the specificity of antibodies from human uveitis patient's sera was first evaluated in two-dimensional (2D) Western blot analysis. Subsequent identification of the immunoreactive proteins by mass spectrometry resulted in the identification of CRALBP as a putative autoantigen. Additionally, sera from human uveitis and control patients were by Western blot using purified human recombinant CRALBP. Anti-CRALBP autoantibodies occur more frequently (P<.01) in human uveitis patients than in normal controls. Thirty out of 56 tested uveitis patient's sera contained autoantibodies reactive against CRALBP, compared to only four out of 23 normal control subjects. The presence of CRALBP autoantibodies in 54% of tested uveitis patients supports CRALBP as a possible autoantigen in human autoimmune uveitis.

## 1. INTRODUCTION

Cellular retinaldehyde binding protein (CRALBP) was recently detected as major autoantigen in equine recurrent uveitis (ERU), a spontaneous model of human uveitis [1]. Similarly to the two other major uveitis autoantigens, S-antigen (S-Ag) [2], and interphotoreceptor retinoid binding protein (IRBP) [3], CRALBP is expressed in both the retina and the pineal gland. CRALBP is a key component of the retinoid visual cycle and participates in the regeneration of 11-*cis*-retinal after photoisomerization [4]. The protein is expressed abundantly in RPE cells, where many reactions of the rod visual cycle take place. Additionally, it is found in Mueller glial cells, which have been implicated in cone visual pigment regeneration [4]. In addition to the RPE and Mueller glial cells of the retina, CRALBP expression has been reported in the ciliary body, cornea, pineal gland, optic nerve, brain, and the iris [4]. While ligands in nonretinal tissues have yet to be identified [4], the protein interacts in the retina with several proteins supporting retinoid supply and participating in the visual cycle [5]. Mutations in the CRALBP gene have been associated with several autosomal recessive retinal pathologies, including retinitis pigmentosa, retinitis punctata albescens, bothnia dystrophy, fundus albipunctatus, and in the newfoundland rod/cone dystrophy [4, 6]. 

Recently, CRALBP was identified as a novel autoantigen in a spontaneous horse disease (ERU), which serves as a model for human autoimmune uveitis. Subsequent characterization of ERU cases revealed B- and T-cell autoreactivity to CRALBP and established a link to epitope spreading [1]. Immunization of experimental animals with CRALBP induced uveitis in two different species with typical tissue lesions at CRALBP-expression sites. ERU shares with human uveitis [7, 8] the autoimmune response to at least two different autoantigens, S-Ag and IRBP. The aim of this study was to investigate the immune response of human uveitis patients to the potential autoantigen CRALBP. 

## 2. MATERIALS AND METHODS

### 2.1. Blood samples and donor eyes

Six normal human donor eyes with no clinical signs of uveitis (2 female, 4 male; mean age 46 years) were used for evaluation of CRALBP expression pattern in normal eyes. Sampling was approved by the local ethics committee in compliance with the tenets of the declaration of Helsinki. All donors gave their informed consent. Peripheral blood samples of 56 patients (34 male, 22 female; mean age 36 years; white Europeans) with uveitis and 23 eye-healthy control subjects (7 male, 16 female; mean age 34 years; white Europeans) were used for this study. Uveitis in patients was defined by intraocular inflammatory signs like endothelial precipitates, cells, and haze in anterior chamber and/or vitreous or signs of retinal vasculitis and choroidal or retinal infiltrates. 

After collection of blood, samples were processed immediately and either serum or plasma was stored at 20°C until Western blots were performed. Donor eyes were fixed 15–60 hours postmortem in 6% buffered formalin. 

### 2.2. Expression and purification of human recombinant CRALBP

Human recombinant CRALBP was expressed in *E. coli* (Invitrogen, Karlsruhe, Germany) and purified on a Nickel agarose column (Qiagen, Hilden, Germany) [9]. Endotoxin was removed on an Endotrap red column (Profos, Regensburg, Germany) and then controlled by a *Limulus* amebocyte lysate test (Sigma, Deisenhofen, Germany; levels below 0.8 EU/mL). The identity of purified recombinant CRALBP was confirmed by mass spectrometry.

### 2.3. Histology and immunostaining

Formalin fixed eyes were embedded in Paraffin (Microm, Walldorf, Germany). Antigen retrieval was performed at 99°C for 15 ⁢minutes⁡ in 0.1 M EDTA–NaOH buffer pH 8.8. We used rabbit anti-human Glial fibrillary acidic protein (GFAP; 1:100, Sigma) antiserum to stain Mueller glial cells and horse anti-human recombinant CRALBP antiserum (1:500) to evaluate CRALBP expression in normal human donor eyes. For fluorescence labeling, GFAP was stained with an anti-rabbit IgG antibody coupled to Alexa 568 (1:500, Invitrogen) and CRALBP with an antihorse IgG-FITC antibody (1:200, Sigma). Nuclei were counterstained with DAPI (Invitrogen).

### 2.4. Two-dimensional (2D) Western blotting

Fresh equine retinas were immediately stabilized with a protease inhibitor (Complete, Roche, Penzberg, Germany), homogenized, lyophilized, and stored at −80°C. For 2D analysis, protein pellets were solubilized in 2D lysis buffer (9 M urea, 2 M thiourea, 1% DTE, 4% CHAPS). Immobiline dry strips pH 3–11 NL, 11 cm (GE-Healthcare, Freiburg, Germany) were immersed overnight in lysis buffer containing 75 μg protein sample, additional 1% pharmalytes pH 3–10 (GE-Healthcare), and 0.5% bromphenole blue. Isoelectric focusing was performed on a Multiphor (GE-Healthcare) for 15 kVh at 20°C, followed by separation on gradient SDS-PAGE gels (9–15%) at constant 45 V per gel. One set of gels was silver-stained for mass spectrometry and the second transferred onto nitrocellulose membranes (GE-Healthcare) for Western blot analysis. Nitrocellulose membranes were blocked with 1% PVP in PBS-T (1 hour) and incubated with sera from uveitis patients and controls (dilution 1:500; overnight at 4°C). Autoantibody binding was detected using rabbit anti-human-IgG-HRP (1:4000, 1 h; Biozol, Eching, Germany) and enhanced chemiluminescence (GE-Healthcare). To assign visible spots to those detected on silver-stained gels, we subsequently stained the nitrocellulose membranes with colloidal gold (Fluka, Deisenhofen, Germany).

### 2.5. 1D Western blots for detection of anti-CRALBP autoantibodies

Purified and LPS-free human recombinant CRALBP (1 μg protein per lane) was applied to 10% sodium SDS-PAGE and blotted semidry onto nitrocellulose membranes. Nonspecific binding was blocked with 1% PVP in PBS-T (1 h). Blots were subsequently incubated with sera in PBS-T (dilution 1:500; overnight at 4°C) washed and then incubated with horseradish peroxidase-conjugated secondary antibody (1:4000; 1 hour, rabbit anti-human-IgG-HRP; Biozol). Signals were then detected with ECL (enhanced chemiluminescence) on Hyperfilm ECL (GE Healthcare) according to manufacturer’s instructions. A monoclonal anti-human CRALBP antibody (0.02 μg/mL; Cayman, Tallinn, Estonia) was used as a positive control.

### 2.6. Image analysis and quantification of signals

Quantification of Western blot signals was performed by densitometry with ImageQuant TL software (GE Healthcare) after scanning the films on a transmission scanner (ImageScanner II, GE Healthcare). Images of blot signals on x-ray films (8 bit/600 dpi resolution) were imported to analysis software (Image Quant TL, v2003) and band volume intensities were quantified. 

### 2.7. Statistical significance

The statistical significance of the frequency of CRALBP autoantibodies in uveitis and control groups was evaluated using the chi-square test. Intensities of positive Western blot signals of uveitis patients and negative controls were compared using the student's t-test.

## 3. RESULTS

### 3.1. CRALBP: a novel human uveitis autoantigen

**Figure 1:**
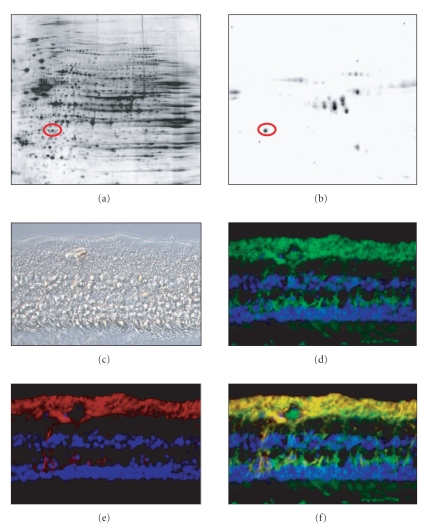
*Identification of CRALBP-autoantibodies with proteomic Western blots.* (a) Equine retinal proteome (pH gradient 3–11, silver staining) was separated on 2D gels and transferred to nitrocellulose membranes. (b) Serum from human uveitis patient detects CRALBP (encircled). *CRALBP expression in normal human eyes.* (c) Nomarski image of normal human retina (donor no. 6, male, age 49). (d) CRALBP expression (green) at Mueller glial cells. (e) GFAP expression (red) at Mueller glial cells. (f) Double labeling of CRALBP (green) and GFAP (red) clearly demonstrates colocalization (yellow) of CRALBP and GFAP at Mueller glial cells.

Autoantibody profiling led to identification of CRALBP as a novel uveitis autoantigen in horses with spontaneous equine recurrent uveitis (ERU) [1]. Sera from human uveitis patients reacted with CRALBP in 2D Western blots using normal equine retinal proteome as a source of antigen (Figure 1(a), equine retinal proteome, Figure 1(b), 2D Western blot incubated with serum of human uveitis patient). The equine retinal proteome was separated by 2D (Figure 1(a), silver staining) over the pH range 3 to 11. Several hundred proteins could be detected as discrete spots using this high-resolution technique (Figure 1(a)). Western blot analysis using sera from human uveitis patients with posterior uveitis followed by mass spectrometric analysis of the immunoreactive 2D gel components resulted in the identification of CRALBP (Figure 1(b), encircled). 

### 3.2. CRALBP is expressed in Mueller glial cells and RPE in normal human eyes

Immunohistochemical staining for CRALBP in six normal human donor eyes (Figure 1(c), Normarski image of donor eye number 6) displayed a strong expression of CRALBP in Mueller glial cells (Figure 1(d), green). To confirm this expression, we additionally stained for glial fibrillary acidic protein (GFAP), a class-III intermediate filament that is Mueller glial cell-specific (Figure 1(e), red). Double staining for CRALBP (green) and GFAP (red) confirmed co-localization of both proteins in Mueller glial cells (Figure 1(f), overlay of CRALBP and GFAP expression yellow) but also CRALBP expression in retinal pigment epithelium. 

### 3.3. Detection of anti-CRALBP autoantibodies in sera of uveitis patients

**Figure 2:**
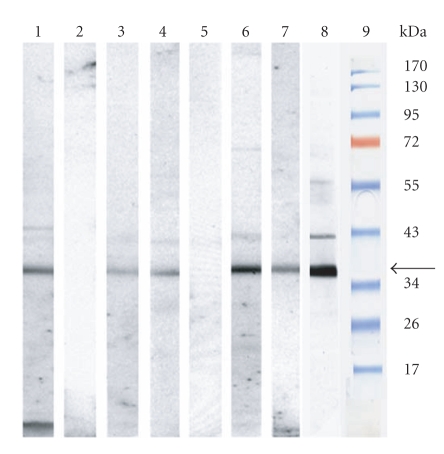
*Representative Western blot signals at 36 kD obtained* *against purified human CRALBP.* Sera of human uveitis patients and healthy controls were tested on 1D Western blots. Lane 1: strong signal of healthy control, lane 2: healthy signal of healthy control, lanes 3 and 4: intermediate signal of uveitis patients, lane 5: healthy signal of uveitis patient, lane 6 and 7: strong signal of uveitis patients, lane 8: monoclonal anti-human recombinant CRALBP antibody (Cayman), lane 9: marker with respective molecular weights.

Since access to fresh human retinal specimen for 2D experiments is limited, we confirmed our findings by Western blots using purified human recombinant CRALBP as antigen. The majority of tested patients was CRALBP-autoantibody positive, however at different levels of signal intensities. Representative results for intermediate, strong, and negative blot results are shown in Figure 2 (signal at 36 kD, intermediate signals lanes 3 and 4, strong signals lanes 6 and 7, negative signals lane 5). A few sera of healthy individuals (controls) also contained anti-CRALBP autoantibodies (Figure 2, lane 1). As positive control, the purified CRALBP preparation was also clearly recognized by a monoclonal human anti-CRALBP antibody (Figure 2, lane 8). 

### 3.4. Significant higher prevalence of anti-CRALBP autoantibodies in uveitis patients

**Figure 3:**
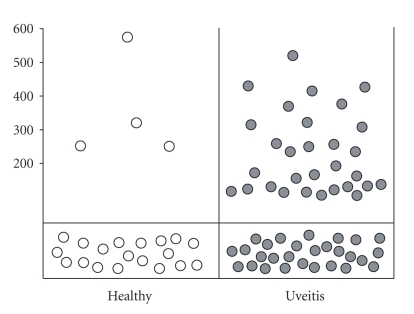
*Frequency and intensity of human IgG autoantibodies* *to CRALBP.* White circles represent healthy individuals, grey circles human uveitis patients. Western blot signal intensities were quantified with Image Quant TL software, grayscale pixel intensity of bands is given as arbitrary units (Y-axis). Negative reactions are dispatched below cutoff line. CRALBP autoantibodies occurred significantly more frequently in uveitis patients as in control group (compared by chi-square test, significance level <0.01).

Thirty out of 56 tested uveitis patients were CRALBP autoantibody-positive (54%) compared to four out of 23 (17%) normal control subjects (Figure 3, white circles represent individual healthy subjects, grey circles uveitis patients). Quantification of Western blot signals and statistical evaluation with student’s t-test revealed no difference in binding intensities between the positive uveitis sera compared to autoantibody-positive sera of normal controls (Figure 3, Y-axis: intensity of Western blot signal as quantified with Image Quant TL software). The higher prevalence of anti-CRALBP autoantibodies in human uveitis sera is statistically significant (P<.01) as evaluated by the chi-square test. 

## 4. DISCUSSION

Most autoimmune diseases display reactivity to multiple autoantigens [10–12]. However, the relative importance of each autoantigen for a specific disease may differ considerably. Autoantigens may appear as a consequence from tissue destruction and thus only represent an epiphenomenon of the pathogenesis, for example, in T-cell-driven diseases. On the other hand, identification of various concurring autoantigens could point to epitope spreading as a pathogenic mechanism in the particular disease [10]. Finally, some autoantigens are useful for predicting individuals at risk for developing the respective autoimmune disease [12]. 

Recently, CRALBP was identified as a novel uveitis autoantigen in horses using a 2D Western blot approach [1]. Reactivity of sera from horses suffering from spontaneous ERU included the known autoantigens S-Ag and IRBP and two novel autoantigens, namely, recoverin and CRALBP [1]. Previously, CRALBP had been described only in association to genetic mutations causing retinal degenerations [4, 6]. Given that CRALBP autoantibodies are present in human uveitis sera (this study) and that CRALBP induces recurrent uveitis in experimental horses and rats [1], CRALBP meets Witebsky’s postulates regarding autoantigenicity [13]. Witebsky originally claimed three postulations to prove a relevant autoantigen. First, the target antigen must be identified; second, an autoimmune reaction against this autoantigen must be detectable; and third, the potential of the autoantigen to induce the respective disease must be proven. Rose et al. proposed three types of evidence to qualify as the third postulate [1]. These are direct evidence from transfer of pathogenic antibody or pathogenic T cells, indirect evidence based on reproduction of the autoimmune disease in experimental animals, and circumstantial evidence from clinical clues [13]. In view of the fact that in this study CRALBP was recognized as an uveitis autoantigen by human patient’s serum and specific autoantibodies were detected in significant amounts in cohorts of uveitis patients (Figures 1(b), 2, and 3), the first two of Witebsky’s postulates are fulfilled [13]. As the uveitogenic potential of CRALBP has already been demonstrated in two different experimental animal species [1], this again meets the Witebsky’s postulates and suggests CRALBP as a true autoantigen for human autoimmune uveitis, accomplishing the third postulate. 

The morphological changes in the retina following CRALBP-induced uveitis in animals show several distinct histological features, which provide researchers familiar with the histopathology of human uveitis a further opportunity to confirm or reject CRALBP as autoantigen in humans from clinical clues (circumstantial evidence). Immunohistochemical evaluation of CRALBP expression pattern in normal human eyes confirmed strong expression in Mueller glial cells [4] through coexpression with GFAP (Figure 1(c)–1(f)). A predominant histopathological finding in CRALBP-induced uveitis in horses was the marked destruction of retinal architecture with rather minor infiltration of inflammatory cells compared to S-Ag or IRBP-induced uveitis [1]. Major changes involved disorganization of Mueller glial cells with fractional destruction accompanied by marked upregulation of glial acidic fibrillary protein and concurrent downregulation of glutaminsynthetase [1], indicating Mueller glial cell proliferation [14]. Additionally, overexpression of VEGF, a major cytokine causing vascular leakage and angiogenesis, could be demonstrated in retinal vessels and Mueller glial cells in CRALBP-induced uveitis in rats and horses [1].

Autoimmune uveitis is a T cell-mediated disease driven by CD4^+^T cells with a Th1 phenotype [15] and can be induced successfully by adoptive transfer of antigen-specific CD4^+^T cells and T cell lines [16, 17]. Therefore, the ability of CRALBP to stimulate T cells of uveitis patients in vitro should be examined. However, a recurring problem of in vitro proliferation assays using peripheral blood derived lymphocytes (PBL) is that responses to autoantigens are rarely detected [8, 18]. Predominantly weak responses to autoantigens were, for example, also reported in the blood of multiple sclerosis [19] or rheumatic arthritis [20] patients. The low frequency of antigen-specific peripheral blood lymphocytes even in advanced cases of uveitis has been discussed as one reason for poor results in these proliferation assays [21]. This hypothesis is underlined by a considerably increased frequency and far stronger response of intravitreal lymphocytes compared to PBL in ERU diseased horses [8]. 

Another aspect of CRALBP autoreactivity in uveitis patients and the few control subjects could focus on the predictive value of this immune reaction. Recent studies in other autoimmune diseases clearly support the potential of foretelling the development of the autoimmune disease when autoantibodies to a number of autoantigens are already present in the preclinical phase [10]. Type I diabetes is one such example and well studied. The presence of autoantibodies against three different diabetes autoantigens has a high predictive value for developing diabetes between 46% and 80% in the general population, which increases to 100% in first-degree relatives [10, 22–24]. It has also been reported that the autoimmune process precedes overt clinical symptoms for many months or years [10]. Therefore, the four CRALBP autoantibody-positive healthy subjects included in this study will be followed on their immune response and health status, since they could be at risk to develop uveitis in the future. Similarly, the CRALBP autoantibody-positive healthy control horses from the first CRALBP detection study [1] are currently monitored. 

A dynamic epitope spreading cascade may exist in spontaneous ERU cases [25] as well as in human uveitis patients [7]. Evidence for the involvement of CRALBP in the spreading cascade was established in ERU cases [1], and therefore CRALBP should now also be included in future studies on epitope spreading in human uveitis patients.

Given that CRALBP function is not fully understood to date [26] and ligands in nonretinal tissues have yet to be identified [4], it is not difficult to assume several yet undetected roles of CRALBP that could be of further interest in association with uveitis. 

## 5. CONCLUSIONS

Our findings suggest that CRALBP is a novel autoantigen for human autoimmune uveitis that merits further investigations.
